# Sentinel Lymph Node Biopsy for Desmoplastic Melanoma: A Systematic Review and Meta-analysis

**DOI:** 10.1245/s10434-026-19498-0

**Published:** 2026-03-23

**Authors:** Peyton Yee, Chengli Shen, Celine Jeun, Mackenzie Mayhew, Russell G. Witt

**Affiliations:** Department of Surgery, University of Virginia Health System, Charlottesville, VA USA

**Keywords:** Melanoma, Desmoplastic melanoma, Pure desmoplastic melanoma, Mixed desmoplastic melanoma, Sentinel lymph node biopsy, Meta-analysis

## Abstract

**Background:**

The propensity of desmoplastic melanoma (DM) to spread to regional lymph nodes remains disputed, creating uncertainty regarding the role of sentinel lymph node biopsy (SLNB). This is further complicated by challenges in distinguishing pure from mixed histologic subtypes. We evaluated pooled SLN positivity rates for both subtypes to clarify the utility of SLNB in the workup of patients with DM.

**Methods:**

Two databases (PubMed and Ovid MEDLINE) were searched through July 2025. We included studies in which at least a subset of patients with DM underwent SLNB and SLNB positivity rates could be ascertained; large registry-based studies were excluded. Our primary outcome was the pooled sentinel lymph node positivity rate for patients with DM. We performed a subgroup analysis to determine pooled SLN positivity by histologic subtype.

**Results:**

We included 18 studies with 1671 patients with DM. A random-effects meta-analysis demonstrated a pooled SLN positivity rate of 9% (95% CI, 7–12%). No significant association was found between positivity and Breslow depth (*β* = 0.058, *p* = 0.749) or ulceration (*β* = 0.763, *p* = 0.677). Ten studies reported subtype-specific rates. The pooled positivity rate was 6% (95% CI, 4–8%) for pure DM and 15% (95% CI, 10–20%) for mixed DM.

**Conclusions:**

Patients with mixed DM may derive greater prognostic and diagnostic benefit from SLNB, whereas SLNB in pure DM may be considered selectively, particularly in the setting of histopathologic uncertainty or other high-risk features.

**Supplementary Information:**

The online version contains supplementary material available at 10.1245/s10434-026-19498-0.

Desmoplastic melanoma (DM) is a rare variant of melanoma, estimated to account for less than 4% of all melanomas.^1^ DM was first described in 1971 by Conley et al., who characterized the variant as “atypical melanocytes and strands of elongated spindle-shaped cells… with abundant dense collagenous substance” that is “highly malignant [and] stubbornly recurring.”^2^ In comparison to nondesmoplastic cutaneous melanomas, DM is more often diagnosed in older males and has a predilection for chronically sun-exposed areas, especially the head and neck.^1,3^ DM can have an inconspicuous appearance, making it challenging to diagnose at the early stages of the disease. It is often amelanotic and presents as an indurated papule, nodule, or plaque, resembling a scar, cyst, or fibroma.^4,5^ Despite its frequent local recurrence and occasional distant spread, nodal metastases appear less common than in nondesmoplastic melanoma.^6^

Desmoplastic melanoma can be subclassified into pure (pDM) and mixed (mDM) histologic subtypes (originally referred to as combined), which have been associated with differing patterns of lymphatic spread.^5,7^ Variability in reported sentinel lymph node positivity rates across studies may therefore reflect underlying histologic heterogeneity as well as inconsistent classification in earlier series.^8^ These uncertainties have contributed to the absence of clear guideline recommendations regarding SLNB in desmoplastic melanoma.

Given the rarity of DM, individual studies evaluating SLNB outcomes are often underpowered, and reported positivity rates remain inconsistent. We therefore performed a systematic review and meta-analysis to determine the pooled sentinel lymph node positivity rate in DM and to evaluate whether SLNB should be recommended.

## Methods

### Search Strategy

**Fig. 1:**
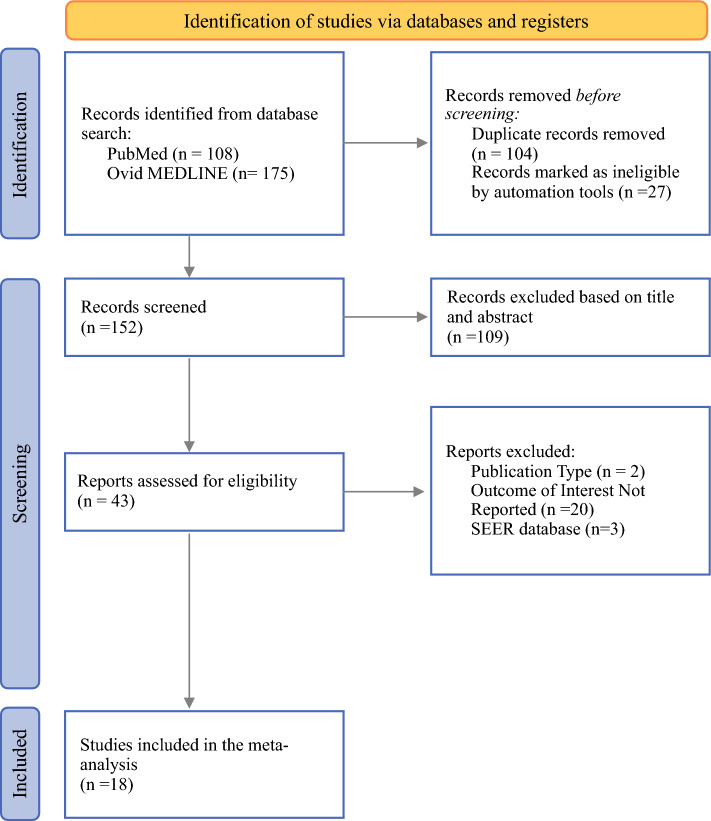
PRISMA flowchart detailing search process. A total of 152 total records were screened, and 18 records were selected for inclusion in the meta-analysis

This review was performed in accordance with the Preferred Reporting Items for Systematic reviews and Meta-Analyses (PRISMA) reporting guidelines and is registered with PROSPERO (ID: CRD420251109333). The review protocol and all amendments, along with justifications, are documented in the PROSPERO record. Two databases (PubMed and Ovid MEDLINE) were searched by one author (P.Y.), and the titles and abstracts of all excluded studies were cross-checked by another author (C.J.). Searches using different combinations of free text (e.g., “desmoplastic melanoma,” “sentinel lymph node biopsy melanoma”) were used to develop and refine our search strategy. The final search strategy, detailed in Appendix A, included free text terms and MeSH terms combined using Boolean operators. The last search was performed on 16 July 2025. The selection process is detailed in the adapted PRISMA flowchart in Fig. 1.

### Eligibility Criteria and Study Selection

We included studies on outcomes of patients with desmoplastic melanoma in which at least a subset of the patients underwent sentinel lymph node biopsy. Studies were excluded if sentinel lymph node positivity rates could not be ascertained. Review papers, studies utilizing large national pooled or registry-based databases, and case reports were not included. Large registry-based studies were excluded *a priori* because they frequently lack granular histopathologic subclassification (pure versus mixed desmoplastic melanoma), standardized pathologic review, and detailed SLNB methodology, which are necessary for reliable subtype-specific pooled estimates. Additionally, studies not published in the English language were filtered out. We reviewed the titles and abstracts of all studies found using the search strategy. After removing studies that did not meet our criteria based on this initial review, the full texts of the remaining studies were reviewed. Studies that met all eligibility criteria were selected for inclusion in the meta-analysis. For the subgroup analyses by histologic subtype, studies were further assessed for stratification of SLNB positivity rates by pure versus mixed DM.

### Data Extraction

We designed a data extraction template that included publication details, sample size, patient demographics, clinical characteristics, and sentinel lymph node positivity rate. One author (P.Y.) extracted the data. For studies that reported outcomes for histology subtypes (pDM versus mDM), we also included the sample size and sentinel lymph node positivity rate for each subtype. All data used in the analyses, as well as template data collection forms, are available upon request from the authors.

### Data Synthesis and Statistical Analysis

A random-effects Generalized Linear Mixed Model (GLMM), assuming a binomial distribution and employing the logit link function, was used for the meta-analysis of proportions. This random-effects approach accounted for anticipated heterogeneity stemming from differences in study populations, methodologies, and geographical locations. Individual study proportions were transformed to the logit scale for the meta-analysis, with the pooled positivity rate and its 95% confidence interval (CI) subsequently back-transformed to the original proportion scale for interpretation. The between-study variance (τ2) was estimated using the Restricted Maximum Likelihood (REML) method. Statistical heterogeneity between studies was assessed using Cochran’s *Q* test (with *p* < 0.10 indicating significant heterogeneity) and quantified by the I^2^ statistic. I^2^ values of 25, 50, and 75% were considered to represent low, moderate, and high heterogeneity, respectively. In addition to pooled proportion estimates, we performed mixed-effects meta-regression analyses to explore whether study-level mean Breslow depth and reported ulceration rates were associated with SLN positivity across studies. Because most included studies did not report subtype-specific covariate data, meta-regression was conducted using aggregate study-level data from the overall desmoplastic melanoma cohorts rather than separately within pure and mixed subtypes. The same GLMM approach was used for subgroup analyses comparing pDM and mDM. All data analyses were performed using R (Version.4.4.0) and the package metafor(4.8). A two-sided *p*-value of < 0.05 was considered statistically significant.

### Quality Assessment

The risk of bias for each study was independently assessed by two authors (P.Y., C.J.) using the Joanna Briggs Institute (JBI) Critical Appraisal Checklists based on study design. Disagreements in scoring were adjudicated by a third reviewer (R.W.). A funnel plot was constructed to visually evaluate the presence of publication bias across the included studies.

### Histologic Subtype Classification

Histologic subtype was defined as pDM or mDM on the basis of criteria reported in the original studies. When specified, strict classification according to the criteria described by Busam et al. was used,^7^ requiring ≥ 90% desmoplastic features for pDM, with mDM defined by the presence of a conventional melanoma component. Studies that did not report subtype-specific SLNB outcomes were included only in the overall pooled analysis and not in subtype analyses.

## Results

### Study Selection

We identified 283 articles through database retrieval. After removing articles involving animals and not published in the English language, we removed 104 duplicate articles. A total of 152 unique articles were screened, and 109 of these articles were excluded based on review of their titles and abstracts. The full texts of 43 articles were assessed for eligibility, and 18 articles were selected for review (Fig. 1).

### Patient Characteristics

Patient characteristics are summarized in Table 1. A total of 1671 patients with DM were included across the 18 articles selected for analysis.^9–26^ A total of 997 patients underwent SLNB, with at least one positive SLN in 95 of these patients. Ten out of the 18 studies included in the analysis reported separate data for pDM and mDM. A total of 570 pDM and 578 mDM patients were included across these studies. In the pDM group, 379 patients underwent SLNB, and 21 of these patients had at least one positive SLN. Alternatively, 53 out of 361 patients with mDM who underwent SLNB had at least one positive SLN.

**Table 1:** Characteristics of patients included in the meta-analysis

Author, Year	No. of patients with DM			median age (years)	median breslow depth (mm)	Ulceration present, *n*(%)
	Total	Pure	Mixed			
Jaroszewski et al., 2001^9^	59	–	–	62.8*	6.5*	–
Thelmo et al., 2001^10^	16	–	–	54.5	3.4	–
Gyorki et al., 2003^11^	32	–	–	64.0	2.2	2 (7.0%)
Su et al., 2004^12^	33	–	–	61.0	2.8	6 (18.2%)
Livestro et al., 2005^13^	89	–	–	63.9	2.6	–
Pawlik et al., 2006^14^	65	46	19	61.0	2.9	8 (12.3%)
Posther et al., 2006^15^	129	–	–	55.2	4.4	28 (21.7%)
Maurichi et al., 2010^16^	242	118	124	64.0	2.0	106 (43.8%)
Murali et al., 2010^17^	252	123	129	60.5	2.6	55 (21.8%)
Mohebati et al., 2012^18^	47	26	18	71.0	6.1*	2 (4.3%)
Broer et al., 2013^19^	24	–	–	64.0	3.0	–
Egger et al., 2013^20^	47	–	–	57.0	2.6	(15.9%)
Han et al., 2013^21^	205	67	61	66.3	3.7	32 (15.6%)
Sims et al., 2017^22^	49	16	19	69.9*	–	9 (21.4%)
Conic et al., 2018^23^	58	15	43	63.0*	3.5*	15 (25.9%)
Laeijendecker et al., 2020^24^	239	114	125	67.2*	4.0	46 (19.2%)
Chu et al., 2021^25^	33	19	14	75.53*	4.6*	4 (12.1%)
Light et al., 2024^26^	52	26	26	76.1*	5.6*	5 (9.6%)

^*^Mean Age/Breslow Depth

### Quality Assessment and Publication Bias

The methodological quality of the included studies was assessed using the JBI Critical Appraisal Checklists. Seven of the 18 studies met all criteria on the checklist. The other studies met most of the criteria, including clearly defined inclusion criteria, consecutive patient inclusion, and appropriate reporting of clinical outcomes; however, a common limitation of these studies was the lack of clarity regarding whether standardized histopathologic criteria were used to diagnose DM and accurately classify its subtypes. Assessment of study quality using the JBI criteria is detailed in Supplementary Table 1.

**Fig. 2:**
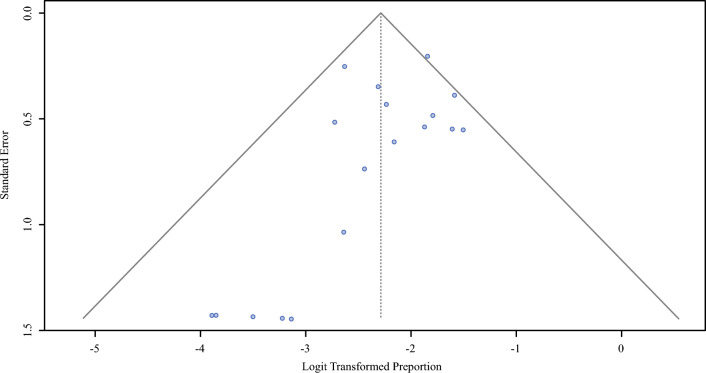
A funnel plot was created to visually assess for publication bias and small-study effects. The plot shows the logit-transformed proportion on the x-axis against the standard error on the y-axis

Visual inspection of the funnel plot (Fig. 2) revealed a relatively symmetrical distribution of larger studies centered around the pooled estimate. Several smaller studies reported low SLNB positivity rates and appeared in the lower-left of the funnel plot. This distribution suggests that studies with small effect sizes or null findings were not systematically missing; therefore, there is no strong evidence of publication bias.

## Study Findings

### Primary Outcome: Pooled Sentinel Lymph Node Positivity Rate

**Fig. 3:**
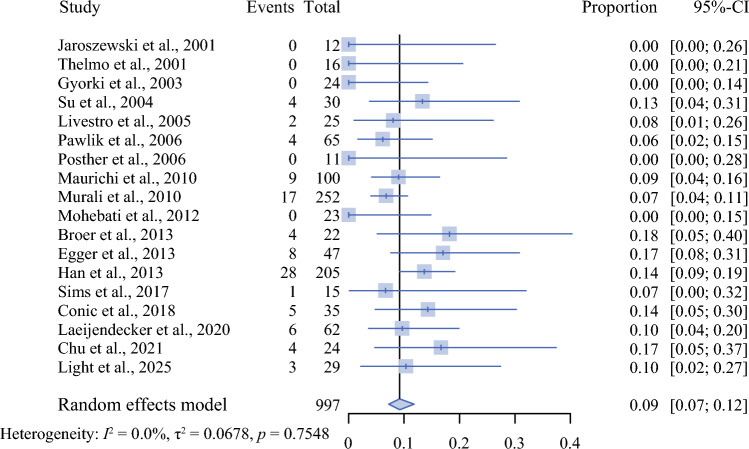
Forest plot demonstrating the pooled proportion of sentinel lymph node positivity among patients with desmoplastic melanoma across all included studies. Individual study proportions are displayed with corresponding 95% confidence intervals. The overall pooled estimate was calculated using a random-effects model. Study heterogeneity was assessed by using the Cochran’s* Q* test and quantified by the I^2^ statistic

A random-effects meta-analysis of all 18 studies demonstrated a pooled SLN positivity rate of 9% (95% CI, 7–12%). Statistical heterogeneity was low, with an I^2^ value of 0% and a nonsignificant Cochran’s *Q* test (*p* = 0.755), indicating consistency across studies and a stable pooled estimate (Fig. 3).

To explore whether study-level tumor characteristics accounted for between-study variability in SLN positivity, we performed a mixed-effects meta-regression using aggregate data from the overall desmoplastic melanoma cohorts (*n* = 13 studies reporting Breslow depth and ulceration, Table 2). Because subtype-specific covariate data were inconsistently reported, regression analyses were not performed separately for pure and mixed subtypes. No significant association was found between sentinel lymph node positivity and Breslow depth ($\beta$ = 0.058, *p* = 0.749) or presence of ulceration ($\beta$ = 0.763, *p* = 0.677), suggesting that these variables do not explain the observed variation in outcomes.

**Table 2:** Study-level mixed-effects meta-regression of SLN positivity in the overall desmoplastic melanoma cohort (aggregate data)

	Estimate	Standard error (SE)	*p*-value	95% Confidence interval
Breslow depth	0.058	0.180	0.749	[−0.294, 0.409]
Presence of ulceration	0.763	1.830	0.677	[−2.824, 4.350]

### Subgroup Analysis: Pure versus Mixed Desmoplastic Melanoma

**Fig. 4:**
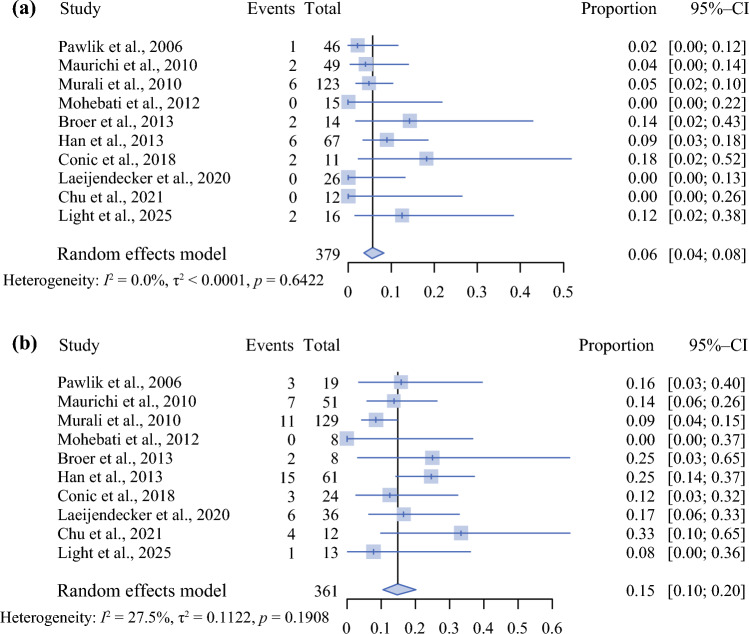
Forest plot demonstrating the pooled proportion of sentinel lymph node positivity among patients with (a) pure desmoplastic melanoma and (b) mixed desmoplastic melanoma . Individual study proportions are displayed with corresponding 95% confidence intervals. The overall pooled estimate was calculated using a random-effects model. Study heterogeneity was assessed by using the Cochran’s *Q* test and quantified by the I^2^ statistic

Given the suggested differences between pDM and mDM, we next performed subgroup analyses to examine SLN positivity by histologic subtype. Ten studies investigated sentinel lymph node positivity in pDM and mDM separately. Among the 10 studies that reported SLN positivity rates by histologic subtype, 7 studies used the 2004 Busam et al. classification system to classify tumors as pDM or mDM.^7^ The pooled sentinel lymph node positivity rate for pDM was 6% (95% CI, 4–8%) with an I^2^ value of 0% and a nonsignificant Cochran’s *Q* test (*p* = 0.642) (Fig. 4a).

The positivity rate for mDM was 15% (95% CI, 10–20%) with an I^2^ value of 27.5% with a nonsignificant Cochran’s *Q* test (*p* = 0.191) (Fig. 4b).

## Discussion

In 2004, Busam et al. classified DM into “pure” and “combined” subtypes, the latter now more commonly referred to as “mixed” DM.^7^ While pDM is characterized by fusiform melanocytes with ≥ 90% stromal fibrosis, mDM has less (10–90%) stromal fibrosis with areas of densely packed tumor cells. These subtypes have been reported to differ in biological behavior, with mDM demonstrating higher rates of lymph node involvement in previous studies.^5,8^

In our meta-analysis, the pooled SLN positivity rate was 6% among patients with pure DM and 15% among those with mixed DM—a threefold difference. When all studies were pooled without distinction between subtypes, the overall SLN positivity rate was 9%, a value likely reflecting the combined inclusion of both pure and mixed lesions. The absence of an association between SLN positivity and traditional prognostic features such as Breslow depth or ulceration further suggests that nodal spread in DM may be influenced more by histologic subtype than by conventional clinicopathologic factors. These findings support the selective use of SLNB in patients with mixed DM, aligning with current practice for other cutaneous melanomas. SLNB provides pathologic staging and prognostic information but does not inherently confer clinical benefit. Although randomized trial data in conventional cutaneous melanoma support potential outcome benefit for SLNB in selected patients with intermediate-thickness disease,^27,28^ comparable evidence demonstrating clinical benefit in desmoplastic melanoma is lacking.

Distinguishing between histologic subtypes is also critical for interpreting the existing literature on SLN outcomes in desmoplastic melanoma. In prior studies, reported rates of SLN positivity have ranged widely: from 0 to 18.2% in pDM, and from 0 to 33.3% in mDM,^9–26^ likely reflecting small samples sizes and inconsistency in subtype classification. Although the majority of included studies applied the 2004 Busam et al. classification system to differentiate between pDM and mDM, 3 of the 10 studies did not.^16,19,24^ For example, one study inferred pDM classification from the absence of mixed features noted in the pathology report.^24^ Variability in diagnostic rigor, as well as differences in anatomic site distribution, may further explain the heterogeneity across cohorts. Standardized histopathologic definitions and consistent reporting of subtype are therefore essential for accurate risk assessment and future guideline development.

Large, pooled databases were not included in our study due to the possibility of overlapping patient populations with several of the institutional cohorts included in this meta-analysis. Several SEER-based analyses have reported markedly lower SLNB positivity rates in desmoplastic melanoma compared with institutional series. In one multicenter study that compared prospective trial data with SEER (1998–2009), the SEER cohort demonstrated an SLN positivity rate of only 2.5% (15/594), far below the 17% rate observed in their combined institutional dataset.^20^ SEER analyses focused specifically on head and neck desmoplastic melanoma have yielded similarly low estimates, with reported SLN positivity rates of 3.7% in a cohort of 244 patients and 5% in a cohort of 165 patients undergoing SLNB.^29,30^ These lower rates likely reflect the inherent limitations of large population-based registries, including heterogeneity in patient selection for SLNB, lack of standardized diagnostic criteria distinguishing pure from mixed desmoplastic melanoma, and variability in the rigor of pathologic examination across reporting institutions. Such differences underscore the need for caution when interpreting SEER-derived estimates in the context of SLNB decision-making for desmoplastic melanoma.

The role of traditional clinicopathologic predictors such as Breslow depth and ulceration in desmoplastic melanoma remains controversial and appears to differ from that in other cutaneous melanomas, particularly once histologic subtype is established. While Breslow thickness and ulceration are well-established predictors of sentinel lymph node positivity in conventional melanoma,^20,26,31–33^ multiple studies have demonstrated that pDM frequently presents with greater tumor thickness yet exhibits substantially lower rates of nodal metastasis compared with mDM and nondesmoplastic melanomas.^14,21,24^ This paradoxical pattern suggests that histologic subtype may function as a dominant modifier of nodal metastatic risk in desmoplastic melanoma, potentially limiting the predictive value of conventional risk factors for SLNB decision-making. Prior studies evaluating Breslow depth and ulceration in desmoplastic melanoma have yielded mixed results and have often focused on survival outcomes rather than SLN positivity.^20,24^ As such, the incremental value of these traditional predictors for SLNB selection in subtype-defined desmoplastic melanoma remains uncertain and is unlikely to be fully resolved without large scale individual patient-level analyses.

The associations between SLN positivity and Breslow depth and ulceration status were assessed for the full cohort using study-level meta-regression, as both factors are independently associated with SLN positivity in cutaneous melanoma.^31^ Given the use of aggregate rather than individual patient-level data, these analyses should be interpreted as exploratory. Subtype-specific meta-regression was not feasible owing to inconsistent reporting of Breslow depth and ulceration within pure and mixed cohorts across studies. In contrast to conventional melanoma, we found no significant association between SLN positivity and either Breslow depth ($\beta$ = 0.058, *p* = 0.749) or ulceration status ($\beta$ = 0.763, *p* = 0.677) for patients with DM. Egger et al. found that ulceration status, but not Breslow depth, was associated with a positive SLNB in patients with DM and that SLN positivity and ulceration were independent predictors of worse disease-free survival.^20^ The discrepancy between these findings likely reflects the limited power and interpretability of study-level analyses in a rare disease, as well as the overall low incidence of nodal metastasis in desmoplastic melanoma and the inclusion of mixed cohorts in which pure and mixed subtypes were not uniformly distinguished. These factors, together with inconsistent reporting of tumor-level characteristics such as Clark level, microsatellites, and mitotic rate, substantially limit the ability of pooled analyses to define reliable clinicopathologic predictors of SLN involvement in this rare melanoma subtype.

The overall methodological quality of the included studies was moderate-to-high based on the Joanna Briggs Institute critical appraisal. Most studies clearly defined inclusion criteria, reported consecutive patient inclusion, and provided adequate follow-up. However, the analysis is limited by the predominance of small, retrospective case series and the absence of patient-level data in many reports, which restricted stratified analyses and reduced the power of meta-regression. Inconsistent histopathologic criteria for diagnosing desmoplastic melanoma and subclassifying pure versus mixed subtypes also contributed to variability across studies. Visual inspection of the funnel plot did not suggest publication bias, and the pooled analyses demonstrated low statistical heterogeneity; however, in the context of rare-event meta-analyses, I^2^ values near zero should be interpreted cautiously, as limited event rates may reduce the power to detect true between-study heterogeneity. Despite these limitations, our study is strengthened by its systematic methodology, adherence to PRISMA guidelines, and use of random-effects modeling with subgroup and meta-regression analyses, providing clinically relevant pooled estimates to inform sentinel lymph node biopsy decision-making in patients with desmoplastic melanoma.

## Conclusions

In this systematic review and meta-analysis, sentinel lymph node positivity differed substantially by histologic subtype, with pDM demonstrating a markedly lower rate of nodal involvement (6%) than mDM (15%). These results reinforce histologic subtype as a key determinant of nodal metastatic risk in desmoplastic melanoma and support a subtype-informed approach to SLNB decision making. While patients with mDM are more likely to benefit from the prognostic information provided by SLNB, a more individualized strategy is appropriate for pDM, particularly when histopathologic classification is uncertain or additional high-risk features are present. Standardized application and reporting of histopathologic criteria will be essential to improving risk stratification and optimizing SLNB use in this rare melanoma subtype.

## Electronic supplementary material

Below is the link to the electronic supplementary material.

Supplementary file1 (DOCX 21 KB)

## Appendix

**Table:**

Database	Search strategy
PubMed	((“melanoma”[MeSH Major Topic] AND (“desmoplastic”[Title/Abstract] OR “desmoplasia”[Title/Abstract]) AND (“sentinel”[Title/Abstract] OR “SLN”[Title/Abstract] OR “SLNB”[Title/Abstract] OR “recurrence”[Title/Abstract])) NOT (“review”[Publication Type] OR “scoping review”[Publication Type])
Ovid medline	(melanoma and (desmoplasia or desmoplastic) and (sentinel or SLN or SLNB or recurrence)).mp. not review.pt.
