# Supplementary material for: Sentinel Lymph Node Biopsy for Desmoplastic Melanoma: A Systematic Review and Meta-analysis
Source: Ann Surg Oncol. 2026 Mar 23;33(7):6719–27. doi: 10.1245/s10434-026-19498-0 (PMC13242384; doi:10.1245/s10434-026-19498-0)
Supplement: Supplementary file 1 — Supplementary file1 (DOCX 21 KB) [file 10434_2026_19498_MOESM1_ESM.docx]

| **JBI Critical Appraisal Checklist for Case Series** | | | | | | | | | | |
| --- | --- | --- | --- | --- | --- | --- | --- | --- | --- | --- |
|  | Were there clear criteria for inclusion in the case series? | Was the condition measured in a standard, reliable way for all participants included in the case series? | Were valid methods used for identification of the condition for all participants included in the case series? | Did the case series have consecutive inclusion of participants? | Did the case series have complete inclusion of participants? | Was there clear reporting of the demographics of the participants? | Was there clear reporting of clinical information of the participants? | Were the outcomes or follow-up results of cases clearly reporter? | Was there clear reporting of the presenting site(s)/clinic(s) demographic information? | Was statistical analysis appropriate? |
| Jaroszewski et al., 2001 | Y | Y | U | Y | Y | Y | Y | Y | Y | Y |
| Thelmo et al., 2001 | Y | Y | U | Y | Y | Y | Y | Y | Y | N/A |
| Gyorki et al., 2003 | Y | Y | Y | Y | Y | Y | Y | Y | Y | Y |
| Su et al., 2004 | Y | Y | U | Y | Y | Y | Y | Y | Y | Y |
| Pawlik et al., 2006 | Y | Y | Y | Y | Y | Y | Y | Y | Y | Y |
| Posther et al. 2006 | Y | Y | U | Y | Y | Y | Y | Y | Y | Y |
| Maurichi et al., 2010 | Y | Y | U | Y | Y | Y | Y | Y | Y | Y |
| Murali et al., 2010 | Y | Y | U | Y | Y | Y | Y | Y | Y | Y |
| Mohebati et al., 2012 | Y | Y | Y | Y | Y | Y | Y | Y | Y | Y |
| Broer et al., 2013 | Y | U | U | Y | Y | Y | Y | Y | Y | N/A |
| Egger et al., 2013 | Y | U | U | U | Y | Y | Y | Y | Y | Y |
| Han et al., 2013 | Y | Y | Y | Y | Y | Y | Y | Y | Y | Y |
| Sims et al., 2017 | Y | N | Y | Y | Y | Y | Y | Y | Y | Y |
| Conic et al., 2018 | Y | Y | Y | Y | Y | Y | Y | Y | Y | Y |
| Laeijendecker et al., 2020 | Y | Y | N | Y | Y | Y | Y | Y | Y | Y |
| Chu et al., 2021 | Y | Y | Y | Y | Y | Y | Y | Y | Y | Y |
| Light et al., 2025 | Y | Y | Y | Y | Y | Y | Y | Y | Y | Y |
| **JBI Critical Appraisal Checklist for Case Control Studies** | | | | | | | | | | |
|  | Were the groups comparable other than the presence of disease in cases or the absence of disease in controls? | Were cases and controls matched appropriately? | Were the same criteria used for identification of cases and controls? | Was exposure measured in a standard, valid, and reliable way? | Was exposure measured the same way for cases and control? | Were confounding factors identified? | Were strategies to deal with confounding factors stated? | Were outcomes assessed in a standard, valid, and reliable way for cases and controls? | Was the exposure period of interest long enough to be meaningful? | Was appropriate statistical analysis used? |
| Livestro et al., 2005 | Y | Y | Y | N | N | Y | Y | Y | Y | Y |

**Supplementary Table 1.** Risk of Bias Assessment

Y = Yes; N = No; U = Unclear; N/A = Not applicable
